# Pulmonary lymphangitic carcinomatosis without concurrent liver metastasis from colon cancer detected using ^18^F-FDG PET/CT: A case report

**DOI:** 10.1097/MD.0000000000017446

**Published:** 2019-10-11

**Authors:** Yueqi Wang, Minggang Su, Lin Li

**Affiliations:** Department of Nuclear Medicine, West China Hospital, Sichuan University, Chengdu City, Sichuan Province, China.

**Keywords:** 18F-FDG PET/CT, colon adenocarcinoma, pulmonary lymphangitis carcinomatosis

## Abstract

**Rationale:**

The infiltration of tumor cells to pulmonary lymphatic system, as known as pulmonary lymphangitis carcinomatosis (PLC), is a rare presentation of pulmonary metastases.

**Patient concerns:**

We reported a case of a 66-year-old man after surgery, chemotherapy, and radiation therapy for colon cancer. Two months after these therapies, the patient complained of nonproductive cough for 1 week.

**Diagnoses:**

18F-fluorodeoxyglucose (FDG)-positron emission tomography (PET)/computed tomography (CT) scanning revealed increased FDG uptake along the thickened bronchovascular bundles, in bilaterally scattered ground-glass opacities and in mediastinal lymphadenopathy. The transbronchial biopsy and pathological study confirmed the diagnosis of PLC.

**Interventions:**

Antineoplastic treatment (cetuximab) were administered after the patient was diagnosed with PLC.

**Outcomes:**

The patient died of respiratory failure within 3 months after the onset of his symptom.

**Lessons:**

18F-FDG PET/CT play an important role in identifying PLC, in selecting possible biopsy sites, and in accessing the extent of metastatic disease.

## 1 Introduction

Colorectal cancer is a major public health problem in China as in many areas of the world. Worldwide in 2012, it resulted in 1.4 million new cases and 694,000 deaths.^[1]^ Colorectal metastases most frequently develop metachronously after surgery for cure. The liver is known to be the most common site of distant metastasis from colorectal cancer,^[2]^ and thus isolated pulmonary metastases are relatively uncommon.^[3]^ On radiographic images, pulmonary metastases most commonly result in spherical, and multiple nodules with different size.^[4]^ Herein, we report a colon adenocarcinoma postoperative patient had unusual features of distant metastasis from colon cancer on 18F-fluorodeoxy glucose positron emission tomography/computed tomography (18F-FDG PET/CT), including involvement of the lungs without liver metastasis and pulmonary lymphangitis carcinomatosis which is the rare form of pulmonary metastasis.

## 2 Case report

**Figure 1:**
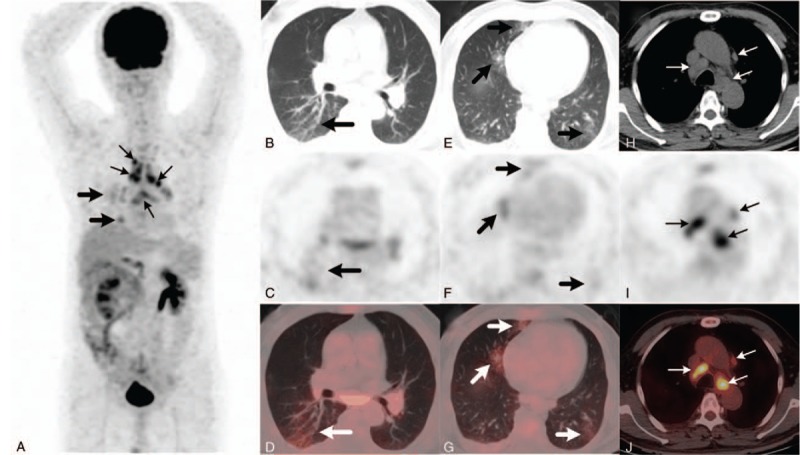
The 18F-FDG PET/CT examination. The maximum intensity projection image (A) showed abnormal FDG uptake in the mediastinal nodes (thin arrows) and right lung (thick arrows). Axial chest images showed, increased FDG uptake in the superior segment of the right lower lobe (B–D) along the slightly thickened bronchovascular bundles (thick arrows, SUVmax=2.7), in bilaterally scattered ground-glass opacities (E–G, thick arrows, SUVmax = 3.7), and in paratracheal and para-aortic lymphadenopathy (H–J, thick arrows, SUVmax, 6.3). 18F-FDG PET/CT = 18F-fluorodeoxy glucose positron emission tomography/computed tomography.

**Figure 2:**
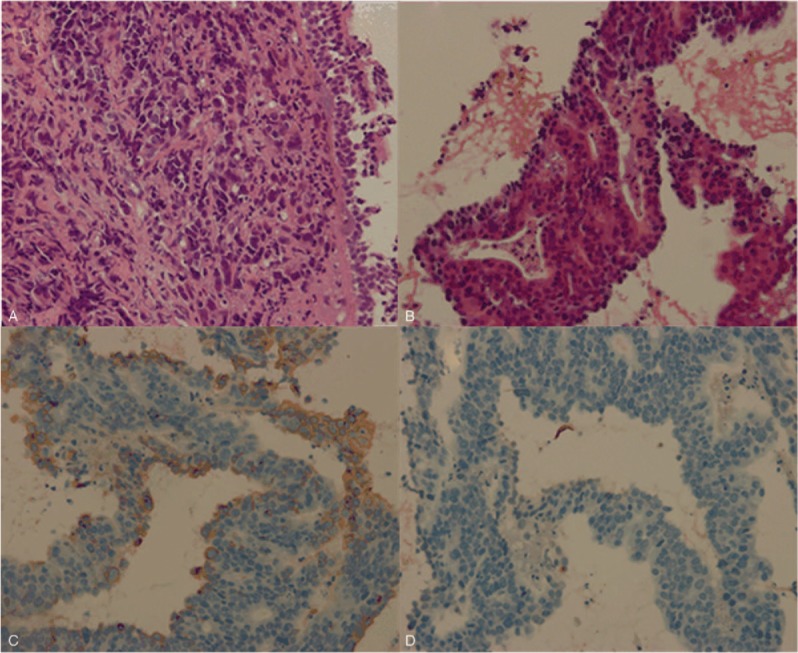
Histopathology revealed that biopsy specimens of the opening of right inferior lobar bronchus (A, hematoxylin-eosin stain; original magnification, × 400) and mediastinal lymph nodes (B, hematoxylin-eosin stain; original magnification, × 400) were composed of marked atypia of epithelialcells. Immunohistochemical staining demonstrated tumor cells were positive for CK20 (C, original magnification, × 400) and CDX-2 (not shown), but negative for CK7 (D, original magnification, × 400). 18F-FDG PET/CT = 18F-fluorodeoxy glucose positron emission tomography/computed tomography.

**Figure 3:**
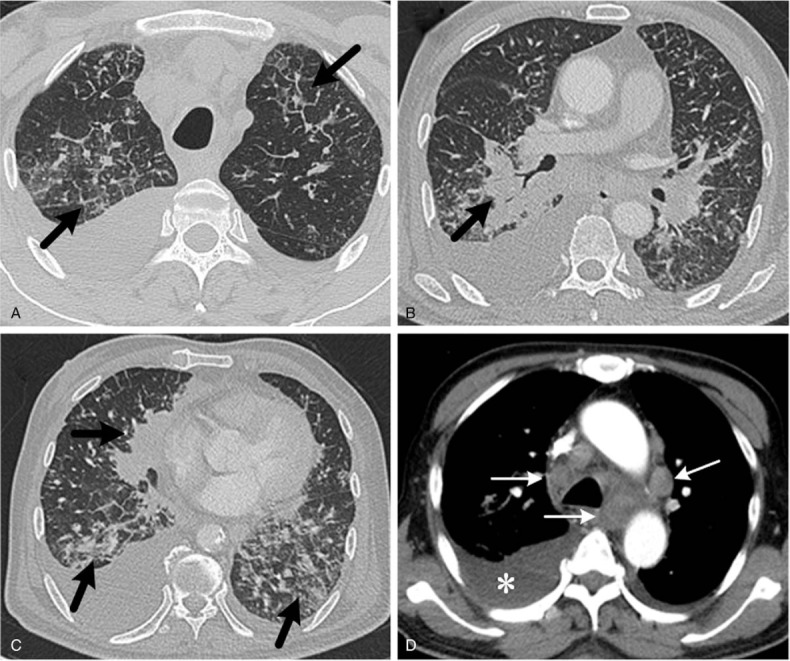
Chest high-resolution computed tomography (HRCT) shows the bilateral areas of diffusely thickened interlobular septa, the thickening of bronchovascular bundles (A–C, thick arrows), the presence of enlarged mediastinal nodes (D, thick arrows), and pleural effusion (D, asterisk). All these features indicate progression of disease.

A 66-year-old man underwent neoadjuvant chemotherapy (XELOX: oxaliplatin and capecitabine) for 4 months followed by surgical resection for poorly differentiated adenocarcinoma in sigmoid colon without lymph node metastases(ypT3N0M0) during October 2016 to February 2017. After surgical treatment, subsequently 3 cycles of concurrent chemotherapy with abdomen radiotherapy were administered. There was no evidence of disease progression after the completion of therapy. In August 2017, he presented to our out-patient department with nonproductive cough for 1 week. He had no fever, chills, hemoptysis, dyspnea, or night sweats. Chest computed tomography (CT) showed bilaterally scattered ground-glass opacities and mediastinal lymphadenopathy. Laboratory test results revealed that the slightly increased serum level of carcinoembryonic antigen, at 4.56 ng/mL (normal range: 0–3.4). Several inflammatory makers, such as C-reactive protein at 97.30 mg/L(normal range: 0–5), interleukin-6 (IL-6) at 48.43 pg/mL (normal range: 0–7), and procalcitonin at 0.17 ng/mL (normal range: 0–0.046), were evidently elevated, considering the possibility of interstitial pneumonia. Given all above clinical symptoms were not alleviated after treatment, he received an 18F-FDG PET/CT scanning for further evaluation, which revealed increased FDG uptake along the thickened bronchovascular bundles (Fig. 1B–D), in bilaterally scattered ground-glass opacities (Fig. 1E–G), and in mediastinal lymphadenopathy (Fig. 1H–J). There was no abnormal FDG uptake in the rest part of the body (Fig. 1A). The patient underwent transbronchial biopsy of the opening of right inferior lobar bronchus and mediastinal lymph nodes. Histopathological and immunohistochemical studies (Fig. 2) revealed that biopsy specimens were composed of marked atypia of epithelial cells (Fig. 2A, B) and were positive for CK20 (Fig. 2C) and CDX-2 (not shown), but negative for CK7 (Fig. 2D). These findings of staining are consistent with metastatic poorly differentiated adenocarcinoma from colon. The patient was transferred to hospital for antineoplastic treatment (cetuximab). In October 2017, he presented to our hospital emergency department with progressive dyspnea. Thickening of the interlobular septa and bronchovascular bundles (Fig. 3A–C), which are the progress of ground-glass opacities, were shown on chest high-resolution computed tomography (HRCT). HRCT also showed further enlarged mediastinal nodes and more serious pleural effusion (Fig. 3D) compared with PET/CT in August 2017. All these features on HRCT indicate progressive disease. Unfortunately, he died of respiratory failure within 3 months after the onset of his symptom.

## 3 Discussion

Sites of distant metastasis are effected by the venous drainage of colon and rectum. Because the venous drainage of the colon is through the portal vein, the liver is a predominant site of distant metastasis, and the concurrent metastasis of liver and lung are more common than only lung involvement.^[5,6]^ On the contrary, the rectum has dual drainage. The middle and inferior veins eventually drain into the inferior vena cava, and this is why lung metastases are more frequent in rectal cancer than in colon cancer.^[3,7]^ Our case points out the relatively uncommon metastatic pathway.

The lungs are common sites of metastases from various malignancies, and PLC is a rare manifestation of lung metastases, accounting for 6% to 8%.^[8]^ The cancer of lung, breast, stomach, pancreas, prostate, cervix, and thyroid are common primary neoplasms to cause PLC.^[4,8]^ PLC has carried a poor prognosis in most cases. Approximately half of patients with PLC die within 3 months after the onset of respiratory symptoms.^[8]^ To our knowledge, there are still no effective anticancer strategies to treat PLC. Only few PLC cases obtained remission by platinum-based chemotherapy^[9]^ and eribulin^[10]^ have been reported. Most reported patients, as we report in our case, received chemotherapy and targeted therapy for primary cancer^[11]^ or just palliative care.^[12]^

PLC can present with dyspnoea and a nonproductive cough.^[11–13]^ HRCT has been the most frequently selected radiographic method for diagnosing PLC. On HRCT, the radiologic features include smooth or nodular thickening of interlobular septa and bronchovascular bundles, ground-glass opacities, hilar and mediastinal lymphadenopathy, pleural effusion, and the still normal lung architecture.^[11]^^18^F-FDG PET/CT images of PLC show increased FDG uptake corresponding to the appearance mentioned above including thickening of pulmonary interstitia and lymphadenopathy seen on the CT,^[14]^ and the mean standardized uptake values of PLC and the normal region of lung are 1.37 ± 0.64 and 0.51 ± 0.29 respectively.^[15]^ Using the ^18^F-FDG PET/CT for diagnosing PLC, Prakash et al^[15]^ demonstrated a sensitivity and specificity of 86% and 100% respectively and diagnostic confidence with this noninvasive method. But the definitive diagnosis should be confirmed by bronchoalveolar lavage^[16]^ or transbronchial biopsy.^[11]^

Although similar symptoms and findings on HRCT and 18F-FDG PET/CT can also be seen on other interstitial lung diseases, such as interstitial pneumonia, sarcoidosis, and cancer-associated sarcoid-like reactions,^[17,18]^ metabolic features of lesions and whole body give valuable information in selecting sites of biopsy, identifying organ involvement not appreciated by routine radiology, and characterizing the extent of metastatic disease. In summary, PLC is a rare but important cause of progressive difficulty of breath and dying of respiratory failure. Rapid onset and progression of dyspnea, FDG-avid thickening of interlobular septa and bronchovascular bundles and hilar and mediastinal enlarged lymph nodes with intense FDG activity in patients with carcinoma of the lung, breast, or stomach should alert us to a diagnosis of PLC. In conclusion, low incidence, atypical clinical symptoms, and nonspecific radiological features are the main causes of the misdiagnosis of pulmonary lymphangitis carcinomatosis. Although the definitive diagnosis depends on pathology, 18F-FDG PET/CT play an important role in identifying PLC, in selecting possible biopsy sites, and in accessing the extent of metastatic disease.

## Author contributions

**Writing – original draft:** Yueqi Wang.

**Writing – review & editing:** Minggang Su, Lin Li.

Yueqi Wang orcid: 0000-0002-1876-0118.

## References

[1] Forman D Ferlay J Stewart BW Wild CP The global and regional burden of cancer. World Cancer Report. France: the International Agency for Research on Cancer, World Health Organization; 2014;p. 16–53.

[2] Fong Y Cohen AM Fortner JG Liver resection for colorectal metastases. J Clin Oncol 1997;15:938–46. 9060531 10.1200/JCO.1997.15.3.938

[3] Mitry E Guiu B Cosconea S Epidemiology, management and prognosis of colorectal cancer with lung metastases: a 30-year population-based study. Gut 2010;59:1383–8. 20732912 10.1136/gut.2010.211557

[4] Davis SD CT evaluation for pulmonary metastases in patients with extrathoracic malignancy. Radiology 1991;180:1–2. 2052672 10.1148/radiology.180.1.2052672

[5] Horton KM Abrams RA Fishman EK Spiral CT of colon cancer: imaging features and role in management. Radiographics 2000;20:419–30. 10715340 10.1148/radiographics.20.2.g00mc14419

[6] Qiu M Hu J Yang D Pattern of distant metastases in colorectal cancer: a SEER based study. Oncotarget 2015;6:38658–66. 26484417 10.18632/oncotarget.6130 PMC4770727

[7] Riihimäki M Hemminki A Sundquist J Patterns of metastasis in colon and rectal cancer. Sci Rep 2016;6:29765. 27416752 10.1038/srep29765 PMC4945942

[8] Bruce DM Heys SD Eremin O Lymphangitis carcinomatosa: a literature review. J R Coll Surg Edinb 1996;41:7–13. 8930034

[9] Kikuchi N Shiozawa T Ishii Y A patient with pulmonary lymphangitic carcinomatosis successfully treated with TS-1 and cisplatin. Internal Med 2007;46:491–4. 17443041 10.2169/internalmedicine.46.6363

[10] Fumet JD Wickre M Jacquot JP Successfully treatment by eribulin in visceral crisis: a case of lymphangitic carcinomatosis from metastatic breast cancer. BMC Cancer 2018;18:839. 30126360 10.1186/s12885-018-4725-7 PMC6102904

[11] Thomas A Lenox R Pulmonary lymphangitic carcinomatosis as a primary manifestation of colon cancer in a young adult. Can Med Assoc J 2008;179:338–40. 18695182 10.1503/cmaj.080142 PMC2492966

[12] Khachekian A Shargh S Arabian S Pulmonary lymphangitic carcinomatosis from metastatic gastric adenocarcinoma: case report. J Am Osteopath Assoc 2015;115:332–7. 25938528 10.7556/jaoa.2015.064

[13] Moubax K Wuyts W Vandecaveye V Pulmonary lymphangitic carcinomatosis as a primary manifestation of gastric carcinoma in a young adult: a case report and review of the literature. BMC Res Notes 2012;5:638. 23158653 10.1186/1756-0500-5-638 PMC3519516

[14] Acikgoz G Kim SM Houseni M Pulmonary lymphangitic carcinomatosis (PLC): spectrum of FDG-PET findings. Clin Nucl Med 2006;31:673–8. 17053382 10.1097/01.rlu.0000242210.99022.fd

[15] Prakash P Kalra MK Sharma A FDG PET/CT in assessment of pulmonary lymphangitic carcinomatosis. Am J Roentgenol 2010;194:231–6. 20028927 10.2214/AJR.09.3059

[16] Meyer KC Raghu G Bronchoalveolar lavage for the evaluation of interstitial lung disease: is it clinically useful? Eur Respir J 2011;38:761–9. 21540304 10.1183/09031936.00069509

[17] Inoue K Goto R Shimomura H FDG-PET/CT of sarcoidosis and sarcoid reactions following antineoplastic treatment. Springerplus 2013;2:113. 23543853 10.1186/2193-1801-2-113 PMC3610027

[18] Chowdhury FU Sheerin F Bradley KM Sarcoid-like reaction to malignancy on whole-body integrated 18F-FDG PET/CT: prevalence and disease pattern. Clin Radiol 2009;64:675–81. 19520211 10.1016/j.crad.2009.03.005

